# 347. SARS-CoV-2 and Acute Otitis Media in Children: A Case Series

**DOI:** 10.1093/ofid/ofab466.548

**Published:** 2021-12-04

**Authors:** Holly M Frost, Thresia Sebastian, Amy Keith, Melanie Kurtz, Samuel R Dominguez, Samuel R Dominguez, Sarah Parker, Timothy C Jenkins

**Affiliations:** 1 Denver Health and Hospital Authority, University of Colorado School of Medicine, Denver, Colorado; 2 Denver Health and Hospital Authority, Denver, Colorado; 3 University of Colorado, School of Medicine, Aurora, CO; 4 Children’s Hospital Colorado, Aurora, CO; 5 Denver Health Medical Center, University of Colorado School of Medicine, Denver, Colorado

## Abstract

**Background:**

Reports in adults with COVID-19 and acute otitis media (AOM) show that severe symptoms and hearing loss may be more common than with the clinical presentation of typical AOM. However, the association of SARS-CoV-2 with AOM in children is poorly understood.

**Methods:**

Cases were identified as a subpopulation enrolled in the NOTEARS prospective AOM study in Denver, CO from March-December 2020. Children enrolled were 6-35 months of age with uncomplicated AOM and prescribed amoxicillin. Children diagnosed with AOM and SARS-CoV-2, detected by polymerase chain reaction assay, were included in the case series. Data was obtained from electronic medical records and research case report forms. Patients completed surveys at enrollment and 5, 14 and 30 days after enrollment that included the Acute Otitis Media Severity of Symptoms (AOM-SOS©) scale. All patients had nasopharyngeal otopathogen testing completed.

**Results:**

A total of 108 patients had been enrolled through December 2020 (all of whom were subsequently tested for SARS CoV-2). During the study period for this case series, 16 patients were enrolled, and 7 (43.6%) were identified with AOM/SARS-CoV-2 co-infection. Among these 7 patients, fever was present in 3 children (29%). Four children (57%) attended daycare. Only 2 children (29%) had testing for SARS CoV-2 as part of their clinical workup. Mean AOM-SOS^©^ scores were similar among the SARS CoV-2 positive and negative patients with no statistical significance noted with two-sided t-tests: 13.6 (± 4.5) vs 14.2 (± 4.9) at enrollment, 1.4 (± 1.8) vs 4.2 (±4.9) on Day 5, and 0.6 (± 0.9) vs. 2.5 (±6.1) on Day 14 (Table 1). Among the 7 patients, no child had an AOM treatment failure or recurrence. Of the 6 patients in whom bacterial and viral testing have been completed, a bacterial otopathogen was identified in 6 (100%), and a viral pathogen in 3 (50%) children (Table 2).

Table 1. Clinical features of children with concurrent SARS-CoV-2 and AOM

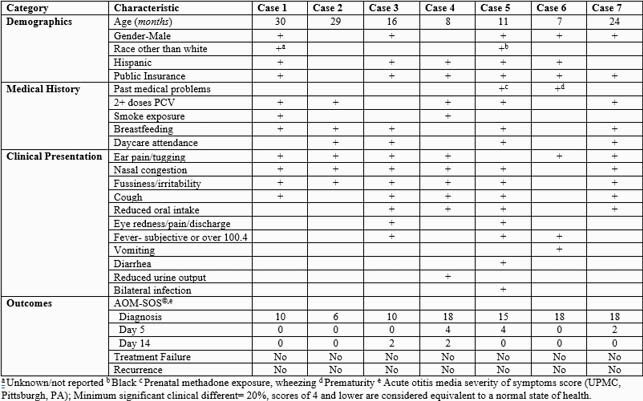

Table 2. Laboratory findings of children with concurrent SARS-CoV-2 and AOM.

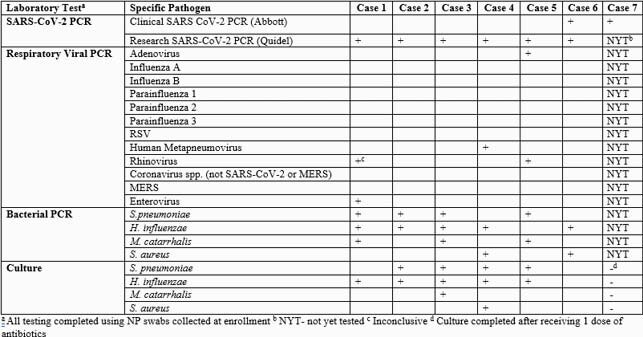

**Conclusion:**

SARS-CoV-2 can occur in children with AOM. It is important that providers maintain a high index of suspicion for COVID-19 even in patients with clinical evidence of AOM, particularly to ensure families are appropriately advised on isolation and quarantine requirements. AOM with SARS-CoV-2 does not appear to be more severe than AOM without SARS-CoV-2.

**Disclosures:**

**Samuel R. Dominguez, MD, PhD**, **BioFire Diagnostics** (Consultant, Research Grant or Support)**DiaSorin Molecular** (Consultant)**Pfizer** (Grant/Research Support) **Samuel R. Dominguez, MD, PhD**, BioFire (Individual(s) Involved: Self): Consultant, Research Grant or Support; DiaSorin Molecular (Individual(s) Involved: Self): Consultant; Pfizer (Individual(s) Involved: Self): Grant/Research Support

